# Weight Loss: A Significant Cue To The Diagnosis of Chronic Mesenteric Ischemia

**DOI:** 10.7759/cureus.5335

**Published:** 2019-08-07

**Authors:** Ayesha Bakhtiar, Adeel S Yousphi, Ali R Ghani, Zain Ali, Waqas Ullah

**Affiliations:** 1 Internal Medicine, Jackson Park Hospital and Medical Center, Chicago, USA; 2 Cardiovascular Medicine, Saint Louis University, Saint Louis, USA; 3 Internal Medicine, Abington Hospital-Jefferson Health, Abington, USA

**Keywords:** abdominal angina, nausea, postprandial pain, chronic mesenteric ischemia, weight loss, diarrhea

## Abstract

Chronic mesenteric ischemia (CMI) is a condition defined by a state of attenuated blood circulation in the mesenteric vasculature affecting one or more abdominal viscera, and is more common in the female and elderly populations. Amongst the many causes, it occurs most frequently in connection with diffuse atherosclerosis. Its presentation is variable with symptoms such as weight loss, nausea, vomiting, diarrhea and/or constipation, with postprandial pain classically present in the majority of the cases; this, in addition to the chronic course of the disease, makes timely diagnosis a challenge. Physical examination may reveal signs of malnutrition and other findings usually linked with the underlying medical condition. It can have grave consequences if not managed promptly. In our case, an 81-year-old woman came in with loss of appetite, nausea, vomiting, diarrhea and/or constipation, and weight loss. Computed tomography angiography (CTA) of the abdomen/pelvis confirmed chronic occlusion of the mesenteric vessels. She was treated surgically. This discussion is based on chronic mesenteric ischemia and its nonspecific symptomatology, particularly its association with weight loss.

## Introduction

Chronic mesenteric ischemia (CMI) can be due to any cause that affects the mesenteric vasculature and compromises blood flow to the abdominal organs, with more than 95% of cases of CMI caused by diffuse atherosclerosis [1]. However, the interconnections between the major mesenteric vessels means the disease does not manifest until two or more vessels are stenosed or obstructed [2]. The chronic course of the disease and the non-specific symptomatology add to the difficulty of diagnosing it timely [3-4]. The disease can give rise to many complications like malnutrition and a decrease in body mass, and can be fatal if not treated, progressing to infarction, necrosis, perforation, sepsis, and death [5]. The following is a case of CMI with a presentation that is not classic for the disease.

## Case presentation

An 81-year-old woman was referred to the emergency department from the nephrology office for low blood pressure. She had nausea and occasional episodes of vomiting, and also reported metallic/salty taste in her mouth and a low appetite which she attributed to the use of spironolactone she had been taking for a month. She had an aortic valve replacement surgery four months back after which she went into respiratory, hepatic and renal failure, and disseminated intravascular coagulation (DIC). The hepatic failure resolved, but she had not been feeling well and had been since living at home with her son who helped her with most of her activities. For the past six months, she had been having alternating constipation and diarrhea and also lost about 40 pounds during this time; however, she denied any fever, dysphagia, odynophagia, abdominal pain, melena, and hematochezia. She also had a temporary episode of facial drooping and swaying to the left side four to five days back, where she did not go to the emergency department but was started on aspirin by her primary care physician. She had a history of chronic obstructive pulmonary disease (COPD), hypertension, high cholesterol, hypothyroidism, paroxysmal atrial fibrillation, hysterectomy, tonsillectomy, adenoidectomy, and cataract surgery. She quit smoking two months back after smoking one pack daily for the past 60 years, and had a glass of wine daily.

On presentation, she appeared thin and cachectic, with a temperature of 98F, blood pressure of 117/65, and a normal pulse, respiratory rate and pulse oximetry. On cardiovascular examination, there was a mechanical aortic valve click and +1 dorsalis pedis pulse. Abdominal examination revealed a bruit on auscultation, positive bowel sounds and there was no organomegaly or any abnormal mass on palpation, while the rest of her systemic examination was unremarkable. On presentation, we suspected medication side effect due to spironolactone as the patient started taking it a month ago, and mesenteric ischemia due to her advanced age, presence of risk factors like smoking, hypercholesterolemia, and hypertension, and symptoms of anorexia, diarrhea, and weight loss.

**Figure 1:**
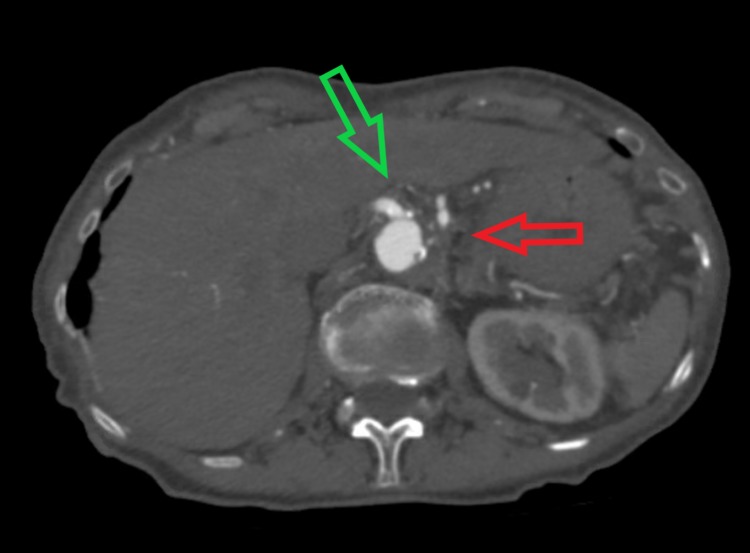
Computed tomography angiography (CTA) of abdomen/pelvis shows occlusion of the proximal superior mesenteric artery (red arrow) and celiac artery (green arrow)

**Figure 2:**
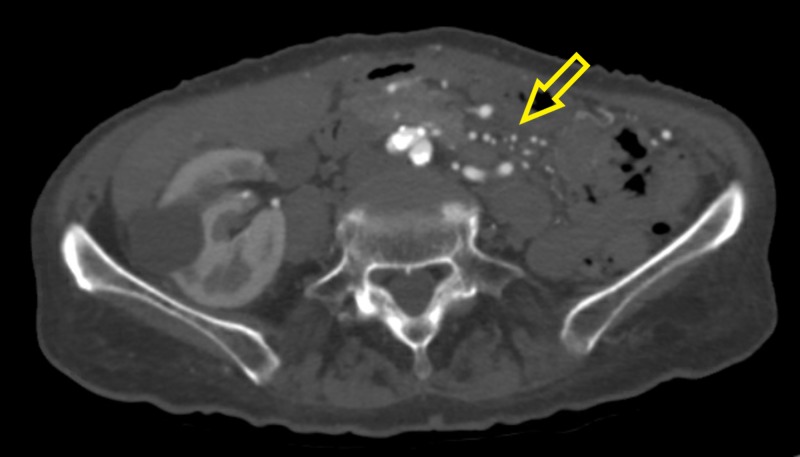
Computed tomography angiography (CTA) of abdomen/pelvis shows inferior mesenteric artery collaterals (yellow arrow)

Pertinent laboratory studies included hemoglobin of 13 g/dL, mean corpuscular volume of 99 fL, platelet count of 282,000/mm3 and white blood cell count of 7.8 x 109/L. Her creatinine and urea nitrogen levels were 0.92 mg/dL and 31 mg/dL respectively. Her serum potassium level was 4.3 mmol/L while serum sodium level was 137 mmol/L, respectively. Her serum protein level was normal at 7.3g/dL, however, her albumin level was slightly low at 3.4 g/dL. Her liver function tests and alkaline phosphatase levels were within the normal range, and electrocardiogram (EKG) showed normal sinus rhythm. Computed tomography angiography (CTA) of abdomen/pelvis showed chronic occlusion of the proximal celiac axis and proximal superior mesenteric artery (Figure 1). The distal portions of the vessels were reconstituted through large inferior mesenteric artery collaterals (Figure 2). CTA also revealed moderate ostial stenosis of the inferior mesenteric artery, and of the bilateral common iliac arteries which were worse on the right side. 

The patient was provided gentle hydration with intravenous fluids and symptomatically treated with ondansetron and proton pump inhibitor. Spironolactone was discontinued because of the metallic taste she was experiencing and borderline blood pressure readings. After the confirmation of the diagnosis of mesenteric ischemia by CTA, the patient underwent successful mesenteric artery bypass surgery. After the surgery, the patient was shifted to the post-anesthesia care unit where she was extubated in stable condition.

## Discussion

Mesenteric vascular occlusion, also commonly called mesenteric ischemia was first described by Allbutt and Rolleston in the latter part of the fifteenth century [5-7]. Mesenteric ischemia is a condition of reduced blood flow which is inadequate to meet the metabolic demands of one or more abdominal organs [8]. CMI accounts for 0.09%-0.2% of all hospital admissions, and can turn out to be life-threatening if not detected and dealt with immediately, with a high mortality rate of 24%-94% [7].

Mesenteric ischemia (MI) can be of two types, acute and chronic MI. Acute MI can be caused by occlusion due to arterial thrombosis/embolism or mesenteric venous thrombosis, or due to low mesenteric blood flow from poor cardiac output or mesenteric arterial vasoconstriction. Chronic MI can have numerous etiologies including vasculitis, malignancy, cocaine abuse, celiac artery compression syndrome (also known as median arcuate ligament syndrome), Takayasu arteritis, radiation, and fibromuscular dysplasia; however, the progressive atherosclerotic disease is the cause in the majority of the instances [5,9]. The population that is at high risk for chronic MI include elderly females aged 60 or above [3-4]. Chronic MI may present as abdominal pain usually 30-60 min after eating and subsequently fear of eating, diarrhea and/or constipation, nausea, vomiting, loss of appetite and weight loss [7-8]. Although a characteristic trait of chronic MI, and being experienced by about 98% of the cases in one study, postprandial abdominal pain can be absent in some cases [10]. Chronic MI can present as nausea, diarrhea and weight loss for a major part of the disease, with abdominal pain occurring at a very late phase in the disease process [6]. Many diseases like peptic ulcer disease, biliary disease, inflammatory bowel disease can give rise to postprandial pain, and therefore it lacks specificity for chronic MI. However, weight loss is a consistent feature of chronic MI [3,7]. Chronic MI can have an atypical clinical presentation, but weight loss along with manifestations of atherosclerosis warrants a strong clinical suspicion of chronic MI [3]. In our case, the 81-year-old female patient did not present with the typical pain, but was suspected to be a case of chronic MI because of existing risk factors such as smoking, advanced age, hypercholesterolemia, and symptoms of nausea, vomiting, diarrhea and constipation, and weight loss. Physical examination may show signs of malnutrition, weight loss, an epigastric bruit, and other findings usually associated with the underlying medical condition; in most cases, signs of widespread atherosclerosis such as transient ischemic disease, as was the case in our patient [8]. Smoking, diabetes mellitus, hypertension, previous vascular surgery and hypercholesterolemia predispose to the development of the disease [11].

Chronic MI is caused by an attenuation in blood flow that occurs over time, and most frequently by the celiac artery, superior mesenteric artery and/or inferior mesenteric artery being narrowed by atherosclerosis [5,12]. These mesenteric vessels form a rich anastomosis, and so in the case of stenosis of a single vessel, the deficiency is made up for by the collaterals. Symptoms mostly manifest when more than one arteries are affected [6]. Mesenteric blood flow normally increases after a meal, but in this case, the blood flow is inadequate to match the metabolic demands of the organs, subsequently giving rise to an inflammatory reaction. This is when most patients confront symptoms of nausea, abdominal pain, fear of eating and weight loss [13]. Chronic mesenteric ischemia can remain asymptomatic until there are typically multiple high-grade stenoses in the mesenteric vasculature, and so is often missed, making it a less common diagnostic possibility [6].

The presence of interconnecting vessels, slow course of the disease, and the lack of specific symptoms make chronic MI a diagnostic challenge. Detection of the disease early in its course mandates a strong index of suspicion. There are various non-invasive investigative modalities for making the diagnosis, including CTA, magnetic resonance angiography (MRA) and duplex ultrasonography. Invasive catheter angiography is another choice to opt for establishing the diagnosis [5-6,8]. CTA, which was used as a diagnostic tool in our patient, is a fast way to scan the entire abdomen and is able to evaluate the collateral vascular system. MRA is another non-invasive modality which can capture detailed images, avoiding the risk of radiation and use of contrast material in comparison to CTA. However, it is potentially difficult to get an accurate assessment of the inferior mesenteric artery with MRA, has a lower resolution and takes longer than CTA, making CTA a more popular diagnostic modality. Duplex US is another imaging modality that has given promising results, but its usage is limited to the screening of proximal artery stenosis or occlusion. Under fasting duplex US examination, a peak systolic velocity of ≥ 275 cm/s for the superior mesenteric artery and ≥200cm/s for the celiac artery indicates ≥70% stenosis. It also necessitates being operated by a skilled technician. Invasive catheter angiography is the ideal investigative modality in the case of chronic MI, and also has a therapeutic advantage, permitting for intervention with angioplasty and/or stenting in the same setting [8,10]. Labs may show anemia, leukopenia, or lymphopenia, electrolyte abnormalities, and hypoalbuminemia as a result of chronic malnutrition [8].

Surgery, which can either be open revascularization (OR) or endovascular revascularization (ER) of the mesenteric vasculature, is the definitive treatment of chronic MI [3,14]. OR involves endarterectomy or bypass grafting, and has a lower incidence of recurrent symptoms, better rate of primary patency and provides symptomatic relief for a longer time [3]. Younger age group or lower-risk patients prove to be good candidates for OR [10]. ER involves percutaneous angioplasty (± stenting), and in comparison, has lower rates of morbidity and mortality, and associated with shorter hospital stays. It is therefore preferred in poor surgical candidates or those with severe comorbidities or short focal lesions [5,15]. Although OR has been the preferred mode of treatment, ER is now being employed more often in the treatment of chronic MI because of the aforementioned factors [8]. Open revascularization with bypass grafting was the therapeutic approach used in our patient, and she was stable thereafter.

Poor surgical candidates are managed with heparin and warfarin to prevent thrombosis/embolism, and in addition, nitrates may provide short term relief. Those who undergo surgical repair need lifelong aspirin, in addition to one to three months of clopidogrel in those with stent placement (unless contraindicated). Lifestyle modifications, and measures such as having small, frequent meals and proton pump inhibitors are only supplementary [6]. Patients need to be managed for risk factors like smoking, blood pressure, diabetes mellitus, hypercholesterolemia, and followed up to ensure optimization of weight and stabilization of nutrition in them [8,10]. Our patient had a successful open revascularization surgery, and her subsequent management plan included control of hypercholesterolemia and high blood pressure, and nutritional rehabilitation.

Chronic mesenteric ischemia can have fatal consequences, but early recognition and treatment can have a significant impact on the overall outcome and prognosis. Malnutrition and considerable weight loss due to the disease pose great difficulties for the patients. It can progress to acute mesenteric ischemia and infarction, perforation, sepsis, and death [8,10]. Although rare, it can recur in up to 17% of the cases, and the likelihood increases amongst the younger age group and amongst those with substantial amount of body mass loss at follow up [16].

## Conclusions

CMI, preferentially affecting the females and elderly, can present with nonspecific symptoms, and can remain asymptomatic until it has progressed significantly, and so its diagnosis requires a high index of suspicion. It should be considered in patients with weight loss, and other manifestations of diffuse atherosclerosis which is a major cause of the disease. It can lead to considerable malnutrition and weight loss, and have fatal consequences such as sepsis and death. Timely diagnosis is associated with a better prognosis, which can be done with imaging or invasive angiography (gold standard). Definitive treatment is with surgery, which can be open revascularization or endovascular revascularization of the affected vessels. Patients need to be followed up to prevent further decline in their weight and nutrition, as well as to prevent the disease from recurring.
